# What photographic portrait to produce to represent Outsider artists?

**DOI:** 10.1017/S2045796023000793

**Published:** 2023-12-20

**Authors:** Lucie Goujard

**Affiliations:** Department of Art History, University Grenoble Alpes, Saint-Martin-d’Heres, France

## From ‘identity’ to the fabulous quest

From the practice of exchanging reproductions of works (Dr Auguste Marie, Hans Prinzhorn and Jean Dubuffet) to the central importance taken by the first illustrated publications, notably that of Prinzhorn, the construction of Outsider Art was therefore eminently photographic. There is a last more remarkable and specific reason: the disclosure of the photographic portrait of the authors of the works has established as a historical ‘event’ in itself. While anonymity is the usual rule in psychiatry and all the ‘artists’ are therefore only identified by a pseudonym or a number, Walter Morgenthaler, keen to organize the best assimilation with art, decides to present, as for him, in a book, the work of his patient, Adolf Wölfli, also disclosing, not without debate, his name and portrait *(Ein Geisteskranker Als Kunstler*…, 1921). It is here that the ‘identity’ is revealed. But was it necessary, nonetheless, to assimilate the series of portraits of him as indisputable witness to his own story? The episode indeed leads to a misleading ambiguity. ‘He reveals the identity of his patient and publishes his photography’ have therefore remained associated ever since in the same historical fact, which collectively suggests that the photographs are simply identity photographs, portraits devoid of any iconographic issue, whereas they are, on the contrary, very obviously imagined. Photographs are thus, here as in other fields, systematically assimilated to indisputable documents, to ‘archive images’. They are received, consequently, as ‘simply informational’ corpora, including when they result from formal constructions and/or contain elaborate narrative and cognitive effects.

The question of iconography has also encouraged another research that has become equally legendary: finding the portraits of the authors. It’s indeed most often an insurmountable difficulty today, either because, being interned, they weren’t photographed or because their lives, generally situated on the margins, didn’t give rise to the preservation of images. Holding the portrait of artists has gradually transformed into a fabulous captive quest to the point of conditioning the historical perception and bringing analysis to the background. An excessively recurrent use of those found has therefore been perpetuated. This, in editions more particularly — scene of a permanent recycling of the same rare photographs, sometimes since the 1920s and 1930s. The portraits, true visual antilogies, ultimately contribute to constructing a quasi-mythological vision of raw artists rather than to providing testimony about them.

## Eloquent portraits. Iconic creation

**Figure 1.**
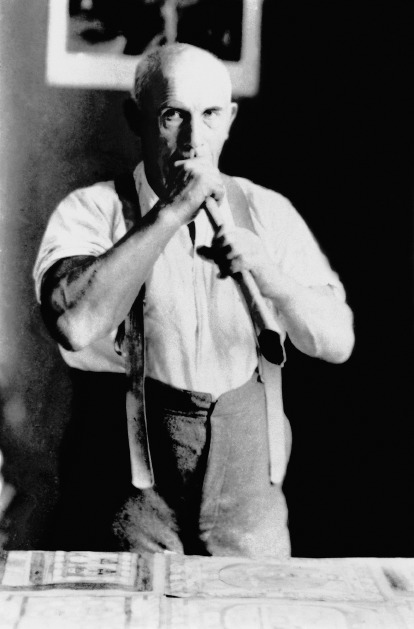
Anonymous.

Exceptional in all aspects, the portrait of ‘Adolf Wölfli with his paper trumpet’ (Museum of Fine Arts of Bern coll.) (Figure 1), the most famous in the series, is characterized, above all, by a powerful and attractive resonance. It was also titled *Wölfli blowing a paper trumpet* (Waldau coll.) and attributed, without certainty, to the psychiatrist Marie von Ries-Imchanitzky in 2022, 10 years after the start of our unprecedented research on this subject. The strength of expression of this portrait is due to the fact that although it gives the impression, nothing is spontaneous there, instantaneous. In fact, we find, on the contrary, clear signs of creative artifices, the elaboration of the pose and expression in particular, as well as the traditional combination and composition of sets and accessories.

*Adolf Wölfli with his paper trumpet*, black and white photograph, photo credit: Adolf Wölfli Foundation, Museum of Fine Arts, Bern; *Adolf Wölfli blowing a paper trumpet*, photo credit: Waldau, Psychiatrie-Museum, Bern. Consult: *Behind the walls. Photography in psychiatric institutions from 1880 to 1935*, exhibition catalog, Musem of Fine Arts, Thurgau, 2022.

**Figure 2.**
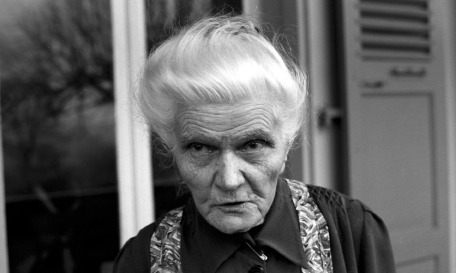
Henriette Grindat, *Aloïse Corbaz, Rosière asylum, Gimel*, 1963.

The challenge and the difficulty of representing portraits in general, appplied here to Outsider Art, is in fact to succeed in thus restoring, in a sensitive and true manner both, the expression, the pose and the gesture of the model. When they also represent his social function, the portraits are, by convention, enriched with the vision of their environment and accessories chosen for their narrative qualities. Attributes, in particular, come into play here. Thus, most often, the artist is photographed or poses in his studio, alongside his works, facing the easel, holding a paintbrush, etc. These portraits therefore ultimately prove to be more the fruit of a desire for (re)fabrication, of their quality as an artist in particular, than a simple recording. Most portraits also focus on describing, restoring them in their ordinary, sometimes ritual life, to the point of reducing their very individuality (Wölfli and his paper trumpet, Aloïse Corbaz and her elaborate hair bun, Augustin Lesage and his miner’s outfit, etc.) (Figure 2). Ipso facto, these different choices therefore profoundly modify the relationship that the reader will subsequently have with the photographs and the impact that they will have on him. They then become not only descriptive but also narrative and cognitive.

The portrait appears on a double page of *L’Art Brut*, booklet 7, by Jean Dubuffet, 1966, entirely devoted to Aloïse Corbaz.

There is a permanent historical aporia between affirmation of the neutral, objective character of the photographs, on the one hand, and narrative production, on the other hand. But although this has been demonstrated by historians of photography, it has strangely been maintained by Outsider Art specialists.

They themselves are seduced by photographic misleading inventions and continue to allow themselves voluntary to be foled by still considering them today, in all contradiction, as ‘archives’ and by basing their theoretical and editorial approaches following this unique documentary consideration of the corpora, however necessarily biased. Thus, the Charm of the visualized History continues, here and elsewhere?
